# Supplementary material for: Causal Relationship between Aging and Anorexia Nervosa: A White-Matter-Microstructure-Mediated Mendelian Randomization Analysis
Source: Biomedicines. 2024 Aug 16;12(8):1874. doi: 10.3390/biomedicines12081874 (PMC11351342; doi:10.3390/biomedicines12081874)
Supplement: Supplementary file 1 [file biomedicines-12-01874-s001.zip › Supplementary Figure/Figure S8/Leaveoneout plot of MR analysis for TL to ubm-b-1635.pdf]

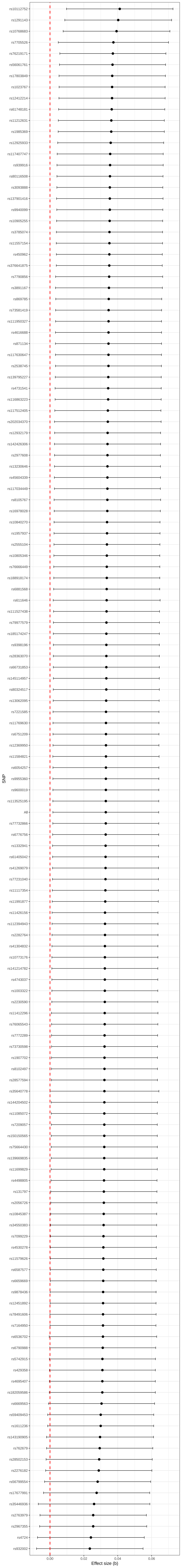

SNP
rs10112752
rs1291143
rs10768683
rs7705526
rs76219171
rs56061761
rs17803849
rs1023767
rs12412214
rs61748181
rs11212631
rs1985369
rs12925933
rs117407747
rs939916
rs80116508
rs3093888
rs137901416
rs9940099
rs10905255
rs3785074
rs11557154
rs450962
rs376641875
rs7790856
rs3891167
rs869785
rs73581419
rs111950327
rs4616688
rs871134
rs117630647
rs2538745
rs139795227
rs4731541
rs116863223
rs117512405
rs202034370
rs12932179
rs142426306
rs2977608
rs13230646
rs45604339
rs117034449
rs8105767
rs16978028
rs10840270
rs1957937
rs2555104
rs10805346
rs76666449
rs188918174
rs6881568
rs611646
rs111527438
rs79977579
rs185174247
rs9398196
rs28363070
rs66731853
rs145114957
rs80324517
rs13062095
rs7221585
rs11769630
rs6751209
rs12369950
rs11584821
rs6054257
rs9955360
rs9600019
rs113525195
All
rs77732866
rs6776756
rs1332941
rs61405042
rs41269079
rs77231040
rs11117354
rs11991877
rs11426156
rs112394943
rs2282764
rs41304832
rs10773176
rs141214782
rs4743037
rs1003322
rs2230590
rs11412296
rs76065543
rs7772289
rs73730598
rs1907702
rs8102497
rs28577594
rs35640778
rs144204502
rs11085072
rs7209057
rs150150565
rs75664430
rs139669835
rs11699829
rs4498805
rs131797
rs2056726
rs10845387
rs34550383
rs7099229
rs4530278
rs11579626
rs6587577
rs6659669
rs9878436
rs12451892
rs78491606
rs7164950
rs6536702
rs6790988
rs5742915
rs429358
rs4695407
rs182059586
rs6669563
rs59409453
rs1611236
rs143190905
rs762679
rs28502153
rs2276182
rs56799554
rs17677991
rs35446936
rs2763979
rs2967355
rs4724
rs932002
0.00
0.02
0.04
0.06
Effect size (b)
